# ‘No-One Can Tell a Story Better than the One Who Lived It’: Reworking Constructions of Childhood and Trauma Through the Arts in Rwanda

**DOI:** 10.1007/s11013-021-09760-3

**Published:** 2021-12-15

**Authors:** Kirrily Pells, Ananda Breed, Chaste Uwihoreye, Eric Ndushabandi, Matthew Elliott, Sylvestre Nzahabwanayo

**Affiliations:** 1 Social Research Institute, University College London, 18 Woburn Square, London, WC1H 0NR UK; 2 School of Fine & Performing Arts, College of Arts, University of Lincoln, Brayford Pool, Lincoln, LN6 7TS Lincolnshire UK; 3 Uyisenga Ni Imanzi, PO Box 7257, Kigali, Rwanda; 4 University of Rwanda, Kigali, Rwanda; 5 Institute of Research and Dialogue for Peace, PO Box 7109, Kigali, Rwanda; 6 College of Education, University of Rwanda, PO Box 55, Rwamagana, Rwanda; 7 School of English, University of Leeds, LS2 9JT, Leeds, UK

**Keywords:** Children and youth, Trauma, Memory, Arts-based methods, Rwanda

## Abstract

The intergenerational legacies of conflict and violence for children and young people are typically approached within research and interventions through the lens of trauma. Understandings of childhood and trauma are based on bio-psychological frameworks emanating from the Global North, often at odds with the historical, political, economic, social and cultural contexts in which interventions are enacted, and neglect the diversity of knowledge, experiences and practices. Within this paper we explore these concerns in the context of Rwanda and the aftermath of the 1994 Genocide Against the Tutsi. We reflect on two qualitative case studies: Connective Memories and Mobile Arts for Peace which both used arts-based approaches drawing on the richness of Rwandan cultural forms, such as proverbs and storytelling practices, to explore knowledge and processes of meaning-making about trauma, memory, and everyday forms of conflict from the perspectives of children and young people. We draw on these findings to argue that there is a need to refine and elaborate understandings of intergenerational transmission of trauma in Rwanda informed by: the historical and cultural context; intersections of structural and ‘everyday’ forms of conflict and social trauma embedded in intergenerational relations; and a reworking of notions of trauma ‘transmission’ to encompass the multiple connectivities between generations, temporalities and expressions of trauma.

## Introduction

Dominant ‘Western’ constructions of childhood and the mind are deeply intertwined. Such constructions are rooted in colonialism, presumptions of universality and underpinned by deeply entrenched power relations between those positioned as adults and those as children, on account of generational and racialised Global North–South relations.^1^ Childhood scholars have traced how the “imagining of the child as imperfect and in a developing stage before adulthood” became naturalised, normalised and exported globally, such as through ‘universal’ models of schooling (Liebel 2020:36) while being “laden with racialised, gendered, classed and sexualised cultural assumptions” about what it means to be a child (Baird 2008:291). Mills and Le Francois (2018) explore how the metaphor of ‘childlike’ has been applied to entire colonised populations, as well as people with mental illness or intellectual disabilities, both historically and in the present. The authors trace how the childlike metaphor is used to “denigrate, to classify as irrational and incompetent, to dismiss as not being knowledge holders, to justify governance and action on others’ behalf, to deem as being animistic, as undeveloped, underdeveloped or wrongly developed, and, hence, to subjugate” (Mills and Le Francois 2018:503). Consequently, certain knowledge as well as ways of being and doing; whether of children and childhood, or of mental illness and manifestations of distress, are privileged over others. This results in the erasure of other forms of knowledge, experiences and practices and the marginalisation of those positioned as ‘other’. Yet as the Rwandan proverb used in the title of this paper suggests: No-one can tell a story better than the one who lived it’.^2^

This article is situated within established critiques of the fields of Global Mental Health (Mills 2014) and humanitarian and postcolonial psychiatry (Fanon 1963; Fassin and Rechtman 2009; Lazali 2021) especially in relation to trauma and Post-Traumatic Stress Disorder (PTSD) (Hinton and Good 2015; Summerfield 1999). Critics argue that in the global circulation of mental health interventions there have been insufficient consideration of the specificity of the origins of such frameworks (Young 1995) nor of the historical, political, economic, social and cultural contexts in which they are enacted (Bracken 2002). We seek to extend these critiques through engagement with critical childhood studies examining the elision between normative assumptions regarding ‘the child’, childhood and the mind (Burman 2019) in order to interrogate power relations (e.g. adult–child, abled-disabled, Global North–South) that sustain such interventions (Akomolafe 2013; Liebel 2020; Mills 2014) and ignore diversity of knowledge, experiences and practices.

We explore these critiques through the case study of Rwanda, where around one million people were killed during the 1994 Genocide Against the Tutsi. We begin by tracing the evolving response to children and trauma in Rwanda from the aftermath of the genocide to more recent concerns with intergenerational transmission of trauma (ITT). We observe continuities in the privileging of certain forms of knowledge and approaches based on psy- frameworks originating in Global North. While such approaches have value, there is the tendency for interventions to be dehistoricised and decontextualised rather than encompassing a broader consideration of the conditions which give rise to and sustain such traumas.

Instead, we heed Mills’ (2014:7) call to think Global Mental Health “otherwise”, in this case from the perspectives of people (children and youth) and spaces (the Global South/Rwanda) positioned as ‘other’. We consider the potential of arts-based approaches to explore other forms of knowledge on trauma, memory and social suffering and to create space for alternative constructions, experiences and praxis in relation to childhood and to mental health. We do so by drawing on the related Connective Memories and Mobile Arts for Peace projects, which used dramatic (image theatre) narrative (proverbs and storytelling), and visual (filmmaking) methods with young people. We conclude by offering some reflections on the need to refine and elaborate understandings of ITT within the Rwandan historical and sociocultural context to encompass the multiple connectivities between generations, temporalities and expressions of trauma.

## ‘Agahinda k’inkoko kamenywa n’inkike yatoreyemo’: Trauma, Childhood and the 1994 Genocide Against the Tutsi^3^

More recent theorisation on trauma and traumatic legacies, especially within the fields of sociology, anthropology and critical psychology, has sought to adopt more expansive understandings of both distress and healing beyond a focus on the individual and the event, to recognise and address external, ongoing sources of distress and how these are rooted in historical and current systemic oppressions, including intergenerational trauma (Hinton and Lewis-Fernández 2011; Kirmayer et al. 2014). In this section we consider these critiques within the context of Rwanda, particularly in relation to the generation born after 1994. In doing so, we take up the question posed by Lazali (2021:2) on intergenerational trauma and Algeria: “how do you inherit a past you never bore witness to”?

The 1994 Genocide Against the Tutsi saw over 1 million mainly Tutsi, but also Hutu opposed to the extremist government murdered in 100 days (des Forges 1999; Mamdani 2002). During the genocide an estimated 400,000 children were orphaned with 110,000 children growing up in child-headed households on account of parental death due to the violence, HIV/AIDS or imprisonment (HRW 2003). Sexual violence was used systematically, leading to a high HIV/AIDS prevalence rate, as well as children born of rape (HRW 2003). Intra-familial and community killings were central to the genocide, severely rupturing the social fabric.

Mental health infrastructure, resources, and personnel, both ‘traditional’ and ‘modern,’ were destroyed during the genocide (Mukamana et al. 2019). In the initial aftermath numerous international organisations, including those focussed on children rolled out psychological assistance programmes, based largely on Western bio-psychological framings of trauma (Mukamana et al. 2019). For example, the UNICEF Trauma Recovery Programme provided counselling for children. However, the service was used by less than one percent of the target population as it was disconnected from the cultural context (Chauvin et al. 2012). Numerous surveys have estimated the prevalence of PTSD and other mental disorders, including among children, typically registering high prevalence rates (Dyregrov et al. 2000; Kayiteshonga et al. 2018; Munyandamutsa et al. 2012; Schaal and Elbert 2006). There has been increasing attention to the well-being of the ‘second generation’ born after 1994, amid concerns of ITT (Berckmoes et al. 2017; Rudahindwa et al. 2020).

While there are examples of approaches and tools being adapted to the Rwandan context (Binagwaho et al. 2021; Bolton et al. 2002) and the development of Rwandan-led policies, programmes and trainings (Ministry of Health 2011), the definitions used are still based on Western psychological approaches (e.g., APA 2013) given enduring unequal global power relations and assumptions regarding the superiority of certain forms of knowledge (Otake 2018; Summerfield 1999; Sinalo 2018). This results in the application of a trauma paradigm that is frequently dehistoricised and decontextualised. (Sinalo 2019).

PTSD is typically conceptualised as a discrete extreme event (APA 2013). While the genocide fits this classification, it does not account for the roots of the genocide in colonial divisionism and post-independence authoritarianism (Sinalo 2019). Rwanda was colonised starting in the 1880s by first Germany and later Belgium. The colonial authorities reconstituted the more fluid social groupings of Hutu, Tutsi, and Twa into fixed ethnic groups, based on fictional origin myths and racial physical stereotypes, initially privileging the Tutsi monarchy, before power was handed to the Hutu majority with independence in 1962 (Prunier 1995).

The post-independence era was characterised by a continuation of the divisionism and exclusion of the colonial period and successive waves of violence, most notably in 1959 and 1973, leading to widespread deaths and exile of Tutsi. As part of the colonial social engineering of Rwandan society, the social institutions of childhood and family were profoundly altered as children were increasingly subject to schooling and the church, with the state assuming a greater prominence in the raising of children. Understandings of childhood were therefore increasingly shaped by so-called universal models of the schooled, developmental child detached from the material realities of children’s lives (Benda and Pells 2020; Boyden 2013). Post-independence, educational quotas restricted the access of Tutsi children to secondary and higher education and poverty presented a barrier for others (Pells et al. 2014).

Thus, rather than viewing the genocide as a single event giving rise to trauma, Sinalo (2018:31) argues that the genocide can be viewed as “a much more complex, chronic form of trauma” encompassing the structural violences of colonialism, neo-colonialism, and authoritarianism, and with enduring legacies in the present. This accords with the work of Fanon (1963) and others (Burman 2019; Lazali 2021) which documents the translation of “colonial trauma” and the dislocation of social and cultural institutions into “social trauma”, leaving an indelible imprint on individual subjectivities and the relations between the individual and society, resulting in further direct and structural forms of violence (Lazali 2021:98–101).

Hartmann et al. (2019) develop a conceptual framework of historical trauma (understood as the collective experience of historical oppressions that result in cumulative effects with cross-generational impacts) as clinical condition, life stressor and critical discourse. It is important to note that this framework is based on a review of studies with American Indian populations and so we are mindful of the specificities of the different contexts in relation to the forms and durations of colonialism, the nature of the genocidal violence and governance in the contemporary era. However, the framework’s conceptual underpinnings and decolonial ambitions provide a productive analytical lens for examining historical and ITT in Rwanda.

Historical trauma as clinical condition adopts a similar symptomology as PTSD. Hartmann et al. (2019) argue that this was intended to achieve recognition for the ongoing impacts of historical violence, such as the Holocaust, other genocides or settler colonialism, on the lives of subsequent generations. For the current generation of youth in Rwanda, historical trauma therefore encompasses the legacies of the colonial and two republics (1962–1994) eras, as well as the events of 1994 and its aftermath. Discussions on ITT are embryonic. ITT does not feature in the Mental Health Policy (Ministry of Health 2011) yet it is a concern attracting increasing attention among policy makers and health professionals, as well as in the media and from survivors’ organisations (Kantengwa 2020). Here, as well as in studies on ITT, the focus remains principally on the genocide and measures PTSD domains (Rudahindwa et al. 2020) or epigenetic pathways (Perroud et al. 2014). Thus, while accounting to a certain extent for the historical context (albeit within in a narrow timeframe) the understandings of ITT and research instruments used are not contextualised.

Even where some cultural adaption has occurred, trauma is still conceptualised as a bio-psychological phenomenon (Otake 2018; Sinalo 2018). This is illustrated by the oft cited Kinyarwanda term ‘ihahamuka’, which is translated literally as breathless with fear and since the genocide has been used in psy- interventions as synonymous with trauma (Taylor 2017). Yet ‘ihahamuka’ does not reflect the complexities of Rwandans’ experiences, understandings, or processes of meaning-making (Denborough and Uwihoreye 2019). Similarly, there is no direct translation of ITT into Kinyarwanda. The term ‘ihungabana mu bana’ meaning trauma in children has started to be used, but similarly to ‘ihahamuka’ does not capture the complexities and nuances of ‘trauma transmission’.

Within Rwandan culture mental distress is conceptualised as arising externally rather than internally to a person within the context of interactions and events (Denborough and Uwihoreye 2019). Ethnographic research has sought to explore localised cultural idioms of distress which are a “way of talking, or other forms of behaviour shared with other people from the same culture…used to express, communicate or comment on distress” (Kirmayer and Gómez-Carrillo 2019:8; Hinton and Good 2015; Pederson, et al. 2010). In Rwanda, idioms of distress are used to describe experiences of suffering emphasise ‘ibikomere’ meaning social and emotional wounds (Otake 2018: 7; See also Uwihoreye, Ndushabandi, and Nzahabwanayo n.d.). Trauma is conceived in social terms and healing understood to occur through social reconnection through everyday life (Otake 2018; Pells 2011).

The second part of Hartmann et al.’s (2019) framework attempts to account for the structural and social context whereby historical trauma is seen as one of many life stressors that compound and impact on subsequent generations. In Rwanda, the social and cultural dislocation wrought by colonialism continues to unfold. Despite rapid economic and social development, the persistence of poverty, difficulties in attending and completing schooling and uncertainties over what is means for youth to be “a good Rwandan” risks reproducing and exacerbating inequalities and impacts profoundly on children’s well-being (Pells et al. 2014). Drawing on qualitative research with 41 families, Berckmoes et al. (2017:4) trace life stressors or “indirect pathways of intergenerational trauma” through which the genocide affects the second generation’s socioecological environment, including poverty, loss of family members, social exclusion, family conflict and parenting challenges.

In addressing historical trauma as life stressor Hartmann et al. (2019:11) suggest that participation in cultural activities or (re-)connections with a strong cultural identity can act as a buffer. Following the genocide, the Rwandan government has sought to re-establish a strong sense of national unity based on the prohibition of ethnic affiliations and the restoration of a common Rwandan identity or “Rwandanness” based on shared cultural values and traditional Rwandan myths and consciousness (Sinalo 2019:170; Ndushabandi 2013). This has been enacted through programmes such as ‘Ndi Umunyarwanda’ (I am Rwandan) and re-establishment of institutions dismantled during the colonial era, such as ‘Itorero’, which provides historical, moral and political education for students who complete high school among others (Nzahabwanayo and Horsthemke 2017; Rutayisire et al. 2017). Critics have expressed concerns over whether such programmes are a form of indoctrination (Longman 2017) whereas others have argued that there remains scope for Rwandans to shape these programmes and debate policy (Rutayisire et al. 2017; Sinalo 2019).

Lastly, Hartmann et al. (2019:11–12) consider how historical trauma can operate as critical discourse or “sociopolitical metaphor” to critique a focus on depoliticised “intrapersonal injury or deficit” such as “problem parenting” to detract from structural factors (see also Fassin and Rechtman 2009 on PTSD). Similar to Cheney’s (2007) research in Uganda, studies on children and youth in Rwanda have highlighted the symbolic instrumentalisation of children and childhood for the purposes of national development (Pells et al. 2014) including contrasting the current generation with the ‘backward’ generation who enacted genocide (Benda and Pells 2020). This is reflected in the Mental Health Policy (Ministry of Health 2011:26) which states that childhood and adolescence are periods of *preparation* for citizenship and *becoming* “a key actor in its development dynamics”. The emphasis here is on children as ‘becomings’, as having value as future actors reflecting normative universalised assumptions, rather than viewing children as ‘beings’ and actors in the present (Liebel 2020). The Mental Health Policy (2011:26) also notes that: “mental suffering in a child is often the consequence of dysfunctional families and societies failure to take its responsibilities”. While there is ample evidence worldwide about the impact of family stress on children’s well-being, the focus often rests on interpersonal dynamics without disentangling how parental trauma, life stressors or the wider sociopolitical environment shapes interactions in the home and processes of meaning-making Kirmayer et al. 2014).

Studies on disclosure practices regarding the genocide have observed both a hesitancy among parents (Berckmoes et al. 2017) as well as a compulsion to share testimonies before children were are exposed to history teaching, peace and values education, which is taught as a cross-cutting subject in schools, or to commemoration activities in the community (Sinalo, Irakoze, and Veale 2020). However, the existence of different narratives in the public and private spheres and the sensitive nature of the social and political environment, with dominant government narratives regarding the past (Longman 2017) can leave parents uncertain about how to talk to children about the past (Sinalo et al. 2020).

In summary, studies in Rwanda have documented ITT as a clinical condition, life stressor and to a lesser extent, as critical discourse. However, despite discussion on intervening with children and youth as groups deemed at risk of ITT, there has been far less focus on how young people make meaning of their experiences and describe suffering and emotional wounds in their lives (for an exception see Denov et al. 2020). In the following sections we consider how cultural forms and arts-based approaches might aid reworkings of understandings of childhood, trauma and ITT, from the perspectives of children and young people.

## ‘Umwana ni umutware’: Arts-Based Approaches with Children and Young People^4^

In the following sections we draw on two arts-based research projects that have been collaborating closely, to explore the connections between forms and expressions of trauma and how these unfold within intergenerational relations.^5^ Arts-based approaches with young people have been aligned historically with critical pedagogy, specifically the work of Paulo Freire. Regarding young people as subjects who “know and act” challenges existing oppressive forms of participation where “knowledge is a gift bestowed by those who consider themselves knowledgeable” (Freire 1996:28/53). In the Sub-Saharan context, there is a long-established a relationship between the arts and popular movements (Kidd and Byram 1982; Mlama 1991; Ngũgĩ 1993).

Art-based approaches, such as Theatre of the Oppressed, have an emphasis on lived experience and have actively contributed to processes of personal and political decolonisation in a range of sociopolitical contexts (Boal 2008; Darder 2015). Notions of dialogue, collective decision-making, and political voice are foundational to youth arts practice to enable young people to address directly matters of concern.

Of course, arts-based methods are not a panacea for decolonising knowledge production as intersecting power inequalities and assumptions, hidden or explicit, persist (Gallacher and Gallagher 2008; Lomax 2012). While both projects were collaborations between partners in the Global North and South, both were funded by bodies located in the Global North which shaped the projects’ designs and timescales, such as having shorter periods of intense workshop activities, to enable those based in the UK to attend. Similarly, the overarching projects were initially designed and led by adults. To navigate the challenges of knowledge construction and power relations, arts-based methods were also employed to reflect on how the projects were running from these perspectives of those involved, such as creating dialogue around any points of tensions and to generate suggestions for adaptations, which resulted in key changes, such as the use of proverbs. Moreover, through young people’s involvement in analysis of images and films, we sought to attend to the multiplicity of perspectives and experiences, whether between young people or between young people and adults (Lomax 2012).

In the second section we reflect on Mobile Arts for Peace (MAP) which began as a pilot project in the Eastern Province of Rwanda*.* Initially, MAP conducted activities to understand the social implications of arts-based approaches within formal (school) and non-formal (cultural organisations) spaces (Breed 2020; Nzahabwanayo 2019). Due to the success of the initial pilot, a further project extended MAP to the remaining four Rwandan provinces and 25 schools. Activities included the filmmaking workshop on which we focus here.

However, we begin by reflecting on Connective Memories (CM). CM is working alongside MAP to adapt the arts-based methods used within MAP to participatory action research (PAR). Working with the same MAP adult and youth trainers acting as co-researchers, we created a PAR project on the theme of ‘isangizanyankuru’ meaning ‘to share stories and memories from your own life or from the lives of others’.

Both sections draw on thematic analysis of observation notes, transcripts of sessions conducted in Kinyarwanda (translated into English), photographs and videos. Both projects received full ethical approval (see declarations) and given the sensitivities of the topics under discussion, had a psychologist or psychosocial worker present, who were available for follow-up with participants afterwards.

### ‘Umugani Ugana Akariho’: Narrative and Image-Based Approaches to Meaning-Making^6^

Connective Memories sought to explore the ways in which young people engage with the concept and practices of memory, in a context where “memory defines trauma, linking current suffering to past events through recollection” (Kirmayer 2015:390). Ten young researchers (aged 12–20) and six adult facilitators used arts-based methods, including image and forum theatre, drawings, photos and films, to create and undertake the research project during 2019. We reflect on two methods; image theatre and proverbs, which were used to create the project.

As noted earlier, given the ways in which the experiences and knowledge of children and young people are often framed by discourses and concepts from the Global North, we wanted to start with concepts and meanings embedded in the Rwandan context and the lived realities of young people. In Kinyarwanda the verb ‘to remember’ (kwibuka) is used to describe the annual commemoration of the 1994 Genocide Against the Tutsi. The group decided that the word ‘kwibuka’ would not be helpful given its close association with official, politically sensitive discourses and would narrow avenues for exploration.

Instead, using image theatre, researchers were asked to create representations of what the idea of memory brought to mind for them. In the first image three adults and one young person formed a bridge-like structure, seated in pairs facing each other, legs and arms outstretched, holding hands, so that their arms formed the bridge. A second young person prepared to step onto the bridge. When discussing their image, the group explained that their image was intended to move away from concepts that associated memory solely with the past (such as ‘isangizanyamateka’ meaning to share history) and instead to see memories as a connected series of events that link the past, present and future. Further, the group suggested that the image indicated the need for support, particularly of the young to overcome struggles, represented by the adults forming the bridge for the young person to cross.

In the second image, two adults and one young person sat on the ground, legs crossed, chin resting in the right hand, gazing upwards to an adult with a ponderous expression, who in turn was gazing down to those seated at his feet. The group elaborated that they wished to illustrate the importance of the context in which memories are shared and how memories are received by those listening, noting that the process of sharing memories may forge or destroy social relations, by connecting to, or silencing, the stories of others.

In the final image, three young people stood in a row. One held out his hands, with a puzzled facial experience. A second held her arms aloft in a sign of victory. A third held her head in her hand, tilted, striking a reflective post. On the ground a young person sat comforting another who had a distressed expression. In reflecting on the image, the rest of the group questioned whether the actors represented a trajectory that someone might pass through following a bad experience. However, the young people responded that the intention was to show how one memory could have multiple emotions and that in listening to the story of another:

you will share their feelings and emotions in some way and then and then the story s/he shares with you will build you as a person rather than just letting him tell you to pass it on to others like that, without personalizing it (young female researcher).

Following the image theatre exercise the group sat together to discuss the ideas generated, how they wished to frame the research project using Kinyarwanda concepts and the questions this topic provoked for them. A series of terms were rejected, such as ‘ihererekanyabuzima’ meaning to transmit stories from one’s life because the group felt that transmission did not encapsulate the experience of sharing in the stories and emotions of others as represented in the images. Eventually ‘isangizanyankuru’ meaning to share stories from your life or the lives of others was chosen as the focus of the project to encompass a sense of connection between the individual and the collective, between past, present and future, and emphasise sharing through multiple modes of expression, beyond the verbal to encompass the embodied and also the symbolic:

When we share, there are links to what people are telling others. This connects between past, present and future. This composes stories that can be sad stories, happy stories and connects to what is a story. A story is something that has happened in the past, present and future. […] When we say isangizanyankuru it means to share with others (adult female facilitator).

The group created a series of research questions, two of which we will reflect on here as these are particularly pertinent to the theme of ITT: what are the challenges of sharing memories? And how can we care for and respect the memories of others? In exploring these questions, the value and significance of proverbs emerged as a means of expressing one’s story and in listening to one another.

Kinyarwanda is a rich metaphorical language and proverbs are one common form of expression.^7^ Proverbs (‘imigani’ plural and ‘umugani’ singular in Kinyarwanda) can be used to represent experiences and signify “intense distress” or other emotions (Bagilishya 2000:341). Proverbs are “often used to express what a person has seen, heard, and experienced at the level of emotions, feelings, and states of mind, as well as to indicate to someone that they have been understood” (Bagilishya 2000:342). In this sense proverbs can open up a conversation or a dialogue, as they call for a response by attempting to elicit “a mode of expression used to recognize, confirm and participate in what the other is living on an emotional level” (Bagilishya 2000:342). Proverbs are also a means of providing advice and guidance, in an indirect or non-judgemental way, while demonstrating an understanding of a person and their situation (Bagilishya 2000). For example, *‘*ibuye ryagaragaye ntiba rikishe isuka’ meaning when you identify a stone in your field, this stone will never damage your hoe, so separating out a problem from an individual. Or ‘umugani ugana akariho’ meaning that the proverb reflects the reality in a concise, clear, complete, and courteous way, showing consideration of the person to whom it is being told.

The young researchers and adult facilitators subsequently conducted a three-day workshop with 20 young participants, aged 12–20. During story circle, part of the MAP methodology, participants are asked to share a story of conflict in the home or community. Participants’ stories were often peppered with proverbs as a means of conveying multiple truths, such as ‘utaganiriye na se ntamenya icyosekuru yasize avuze’ meaning when you do not talk with your father, you cannot know what your grandfather said before dying. This can be interpreted literally in the sense of regret at lack of family communication but takes on added significance in Rwanda with the often near absence of entire generations within families because of the genocide and its legacies. Proverbs were also used to frame sessions focussed on the challenges of sharing of memories, such as ‘Ikinu kibi kibaho ni ukubwira utakumva’ (it is hurtful is to talk to someone who is not interested) or ‘kubwira utakumva ni nko guta inyuma y’umusozi wa huye’ (talking to someone who is not hearing you, is like to throw far beyond Huye forest).^8^

This approach resonated with participants. In a reflective exercise where participants were asked which moment from the day, they were going to take away with them, many repeated one of the proverbs that had been shared and with which they had connected. Participants observed that the expression of a proverb after a story has been told, was a means of expressing an appropriate emotion, so honouring the person and respecting the memories of others to answer the question posed by the young people.

Proverbs therefore are a familiar part of the Rwandan cultural landscape and offer a way of exploring and responding to experiences of suffering in a contextually appropriate manner. This resonates with the work of Hartmann et al. (2019), Hinton and Good (2015) and Kirmayer et al. (2014) among others, on how (re-)connection with cultural forms can aid healing processes, especially in contexts where there have been long histories of cultural dislocation and dehumanisation. Proverbs also re-centre the person telling the story as the expert: ‘Ntiribara umukuru nk’umuto waribonye’ (meaning adults cannot explain better an event than the young person who has experienced it) which is particularly pertinent for challenging adult–child power relations and the ‘othering’ of children as lacking in knowledge. Young people are therefore recognised as the experts of their own lives and experiences and dignified by using ideas and language with which they are familiar, thus responding to some of the shortcomings of approaches outlined earlier.

### Mobile Arts for Peace and Mobile Filmmaking

In the MAP project, young people identified everyday conflicts and opportunities for peacebuilding enabling connections between “everyday” forms of trauma (Craps 2013:27), social conflict and social healing (Otake 2018) to be explored. Rwandan producer and director Eric Kabera conducted a three-day workshop focussed on filmmaking using mobile phones, working with the same ten MAP youth trainers and six MAP adult trainers.^9^ The role of film in peacebuilding has been described as a process that can “bridge the gap between state-centric and grassroots approaches by bringing attention to the agency, needs, customs, and mobilization of individuals and local communities” (Townsend 2016:29). In this way, as noted earlier, art and engagement with cultural activities can act as a mediator between state-driven narratives concerning conflict and peace and locally produced knowledge situated within everyday encounters and events (Hartmann et al. 2019).

“What stories can you tell from the perspective of an object?”, Kabera asked the group at the start of the workshop. He took off his shoe and told a story about some of the journeys that his shoe had been on. Kabera stated:

Your strongest tool is not the camera; it is your eyes and the power of observation. Filmmakers are storytellers. Storytellers are able to see what others can’t see. It is important to incite curiosity and to see things in new ways.

The adult and youth trainers created working groups of between four to five members to create a short film from the perspective of an object that contained a moral or message relating to issues in their community. The trainers wrote, directed and acted in the films, which were premiered at the 2019 Rwandan Film Festival.

A follow-up analysis workshop was conducted online via Zoom in January 2021. Some of the discussion questions included: What was the story? What were you trying to say? What were your aims? How was the story being told through the perspective of an object/person/place? What was the main theme of the film? If you were to continue developing the films, what would you do? We first watched the films together and analysed the films based on the discussion questions. On the second day we watched the films together and then analysed the film by stopping and starting the film whenever a participant would call out ‘hagarika’ (stop in Kinyarwanda). This technique followed the dialogic approach of MAP to bring participants into dialogue with the art form and content of the films. Next, we provide an overview of each film in relation to the analysis provided by the six adult MAP adult trainers and four MAP youth trainers who attended the analysis workshops.

#### The Plate at School (2019)

The story emerged from requirements for every student to bring a plate from home to school. According to the participants, this requirement has caused conflict between teachers, school administrators and students. Numerous reasons were cited in terms of how and why students might not bring plates to school including embarrassment or shame over not having a plate or only a poor quality plate (illustrating economic disparities between students); and resistance to authority.

A youth female trainer stated: “When we were making the film, we wanted to show that bad behaviours originate from simple things. It can lead to bigger mistakes. From minor things kids or children can become addicted to wrong things; that is why we used a simple thing to show how it can lead to committing big thoughts that lead to negative things.” Here, there is attention to everyday conflict; that small conflicts can lead to bigger conflicts as children are caught navigating discourses of what it means to be a ‘good child’ within the material constraints of their everyday lives (Pells et al. 2014). Another adult male trainer stated:

In the beginning of the film, it is the start of term. The rules have been set. Students didn’t get a chance to share their opinions. Schools set regulations without student input. Rules only to execute aren’t fair.

This extends the analysis beyond behavioural explanations to explore the root causes of the conflict based on lack of student input and hierarchies of power that do not engage children in decision-making processes. Additionally, the reflection also brought out issues related to mental health and well-being. One trainer stated that students are punished for not bringing their plate to school by not being fed. A suggested alternative was to have a school counsellor to speak to those who did not bring their plates to school to understand the issues that prevented them from doing so. It was discussed how more open communication could help alleviate some of the conflict. However, it was notable that there were variances between the narratives of young people (focussed on hardship and distress) versus adults (focussed on regulations). In particular, the young people were actively engaged in the discussion through the chat function of Zoom to offer alternative explanations, which may illustrate the potential benefits of using technical platforms for research, due to unequal power relations between young people and adults and for digital platforms to provide opportunities for young people to express themselves.

#### Telephone (2019)

This story illustrated everyday conflicts in relation to the high demand and cost of mobile phones in Rwanda alongside family conflicts that can be created by material objects. Participants noted additional issues represented in the film included gendered power inequalities and the need for support to assist individuals and families in communicating to enhance family well-being. A young male trainer stated: “The film portrays that anything that is used in a wrong way can cause harmful things; can cause bad things versus serving its purpose.” Another adult male trainer stated:

Our aim in making the film was to inform the community that when they decide to buy any tool or communication like telephone or electronics that they have to be prepared that from that, conflicts can be raised. It is their duty (a must as a member of family) to sit and discuss how to use tools wisely and how to solve conflicts if there is a misunderstanding due to the use of the tool; how they can find a solution.

Here again, there was an emphasis on the need for dialogue and communication. Young people observed that adults are often too busy with their phones to listen to the needs and issues of young people. This underscores the importance of not only seeing expressions of distress as being related to the past but connected also to concerns with contemporary rapid social and economic changes, linked in part to globalisation (Pederson, et al. 2010).

#### Headphones (2019)

The story focuses on a pair of headphones that a young person drops while walking from a restaurant to his home with his mother. Another young person finds the headphones and chases after the family to return the found headphones. This follows with an altercation between the owner of the headphones and the young person who found the headphones, as the owner initially blames the person for stealing them.

The participants commented on the ongoing and underlying lack of trust in communities and the need to build trust and communication. As one adult female trainer stated: “after taking time to listen, then come up with a decision. Don’t be aggressive; pay attention to everything and then decide what to do. In society, be careful, listen to a person.” Another male trainer asked the filmmakers: “Why did you chose to focus on the headphones instead of a phone that has more value?” In response, an adult female trainer stated that they wanted to focus on something small to emphasise that the expression of values within interpersonal relations on a micro level will inform societal level issues. Although there was initially mistrust between the owner of the headphones and the person who found the headphones, it was important for the artists to emphasise that the effort to return the headphones was valued. One youth trainer stated: “We wanted to show the emotions. There is a close up on the person who returns the headphones. He brings headphones with love; he didn’t think it would cause a conflict.”

#### Amazing Dog (2019)

The story is narrated from the perspective of a stray dog that is never actually seen on camera, but is portrayed through sounds, camera angles and dialogue. Participants noted that dogs are not valued in Rwandan society and are rarely kept as pets. In the film, a young person finds a dog and brings it home. There is conflict between him, his sister and his mother. However, the father recognises the love and affinity that his son has with the dog and they discuss the benefits of having the dog as a family.

Participants highlighted the importance of discussing issues as a family to come to a common understanding and to avoid conflict. A female youth trainer stated:

The reason that we made our film was to show family conflicts. The choice was the dog; a simple thing; but no family members had the same ideas. We wanted to show that there are some things that kids love; does nothing bad in the family. The dog was lovely and amazing and was finally admitted to the home.

Another female youth trainer emphasised the role of dialogue in solving conflict: “Sometimes conflicts are solved through dialogue. The father and mother discuss the behaviour of their son, then accept. The dog is amazing for all of them.” By listening to the child and valuing what they loved, it led to positive change within the family.

Through the films and discussion workshops participants expressed the importance of considering everyday conflict and trauma in order to understand and to address wider social conflicts or trauma, such as an enduring lack of trust in communities post-genocide (Headphones) and the importance of sharing stories and building dialogue to address sources of distress and conflict (Nzahabwanayo 2019). While these discussions build on insights from studies conducted with Rwandan adults on the need for approaches that attend to the collective familial and community context for healing (Otake 2018; Sinalo 2018) the films also highlighted differences in perspectives between adults and young people and the need to consider intergenerational tensions. Power relations between adults and children were central to the film narratives, whether between children and parents (Amazing Dog and Telephone) or students and teachers and were shaped by life stressors experienced by poor families, such as the tension between national policies in relation to schooling and the material realities of children and families’ lives (The Plate at School). Equally, the processes of filmmaking, as well as the films themselves, offered possibilities for intergenerational collaboration and dialogue, demonstrating the values of the arts in creating spaces for alternative stories and moral imaginings to emerge that move beyond narratives of victimhood to consider individual and social flourishing (Hartmann et al. 2019; Kirmayer et al. 2014).

## ‘Urwishe umukondo ntirusiga ubura’: Conclusions^10^

In conclusion, how might arts-based methods assist us in thinking “otherwise” about childhood and Global Mental Health, particularly ITT (Mills 2014:7) from the perspectives of those positioned as ‘other’ both as children and from the Global South? The framework of historical trauma and ITT as clinical condition, life stressor and critical discourse is a helpful departure point for exploring the experiences of the Rwandan generation born after 1994. However, using arts-based methods we argue that there is a need to refine and elaborate understandings of ITT informed by: the Rwandan historical and sociocultural context; intersections of structural and ‘everyday’ forms of conflict and trauma embedded in intergenerational relations; and reworking of notions of trauma ‘transmission’.

Regarding Historical Trauma or ITT as clinical condition, it is important to note neither CM nor MAP are clinical interventions. In the wider literature, the evidence on ITT in Rwanda as clinical condition is mixed, with some studies finding effects (Perroud et al. 2014) others not (Roth et al. 2014) and others finding differences between population groups, such as between children of survivors and of prisoners (Rieder and Elbert 2013). This highlights the danger of “biological reductionism” as bio-psychological explanations can only provide a partial picture of ITT (Kirmayer et al. 2014:309), can perpetuate a coloniality of knowledge production and obscures “the intense interpretative activity that reflects culture and politics as much as human biology” (Kirmayer 2015:389). Echoing Kidron’s (2009) research with adult descendants of Holocaust survivors the CM project highlights how memories, and the sharing of memories, are embodied, affective and relational, and may be expressed through the symbolic, metaphoric and non-verbal, including objects and silences, as well as through verbal and narrative form (see also Kirmayer 2015).

Both projects demonstrate the value of cultural forms in engaging young people in ways that are meaningful and resonant with their own experiences (Uwihoreye et al. n.d). The use of metaphors (whether visual or verbal) enabled sensitive topics, such as memory, conflict or inequitable generational and gendered power relations, to be explored indirectly, when open expression might not be possible (Kirmayer 2015). For instance, the plate served as a metaphoric object to demonstrate economic disparity between students and the disconnect between the symbolic value and high aspirations attached to schooling by families living in poverty on the one hand and the myriad barriers on the other (Boyden 2013; Pells et al. 2014). In this way, objects provided a means to explore everyday conflicts alongside potential solutions through intergenerational dialogue. Similarly, the metaphoric language of proverbs enabled the expression of difficult stories or memories, but crucially, also attended to the response of the ‘listener’, focussing on listening as an embodied act and a way of dignifying the ‘teller’, demonstrating care and respect for their story through the (re-)connection with cultural heritage (Bagilishya 2000). Cultural forms therefore offer a rich resource to expand understandings of ITT, contextualised in the Rwandan context and from the perspectives of young people, rather than imposing external discourses or interventions.

When considering ITT as situated among multiple life stressors, the MAP films highlight the value of adopting a Fanonian approach to trauma and distress that connects the intrapersonal, interpersonal and structural (Fanon 1963). Both projects hint at the intersections between everyday, structural and intergenerational forms of trauma and conflict. For instance, the films illustrate how longer-term structural inequalities emerge in everyday forms of conflict and sources of distress. Similarly, the young researchers reflected on how adults often do not share stories or memories with them “because many of us, the young, we were not there in the past events of the country they told us we do not understand” (young female researcher) reflecting widespread societal assumptions about children and the status of childhood.

This raises a couple of questions for future exploration. First, how are historical and everyday ‘wounds’ intersecting to shape children’s lives and well-being? While each generation’s struggles may be tied to that of previous generations, we should not overlook commonalities in experience (e.g. parents also inherited the traumatic legacies of colonialism and the post-independence waves of violence) nor assume that these struggles manifest in the same form, accompanied by the same life stressors in the contemporary context, or the same configurations of intergenerational power dynamics (Kirmayer et al. 2014).

Second, how are young people making connections between everyday, structural and historical forms of trauma? Other studies suggest that young people are constantly navigating official narratives, discourses and programmes, within their everyday lived realities (Pells et al. 2014; Benda and Pells 2020). This underscores the need for further work to disentangle the distinctive “looping effects” from “ideology and policy to structural, institutional, and interpersonal violence (and back again)” as experienced by Rwandan youth (Kirmayer et al. 2014:307, 313). In addition, this suggests the caveat that when drawing upon arts-based methods to explore life stressors, it is also important to consider the possible “limiting effects on how the causes of distress are framed and how redress is sought” by focussing on cultural rather than social and economic materialities (Kirmayer et al. 2014:311). The latter being crucial in shaping the research process, as well as which stories are heard and which are silenced (Lomax 2012).

Finally, considering ITT as critical discourse requires not only interrogating predominant framings of trauma, but also of constructions of childhood that position children, particularly in the Global South as ‘other’ or incompetent (Mills and Le Francois 2018). Using arts-based methods, children and young people challenged the notion of ‘transmission’ whereby children are positioned as the objects or subjects of ITT or of knowledge production, rather than considering children as generators of knowledge and active translators and mediators of individual and social or collective memory (Kidron 2009; Pells 2018). This challenges the future-orientated ontology of the child as developmental ‘becoming’, typically embedded in national-level policies and discourses of ITT. Instead, we suggest that understandings of ITT need to encompass multiple (rather than linear) temporalities, which unfold within intergenerational relations, as illustrated by the CM images like the bridge or the affective co-presencing from different temporal moments represented in the second and third images (Mbembe 2001). A shift from focussing on ‘transmission’ to multiple ‘connectivities’ would enable a deeper appreciation of the multidirectional linkages between generations; between intrapersonal, interpersonal and structural forms of trauma; between temporalities, and between multiple modes of expression. More widely, this shift would facilitate questioning of which social and institutional structures enable or constrain the possibilities for intergenerational encounters and the sharing, rather than the transmission, of knowledge and experiences, in order to think ‘otherwise’ about childhood and trauma.

## Footnotes

1 ‘Global North–South’ is used to broadly characterise global inequities in the distribution of power, resources and knowledge production, while recognising the problematic, reductionist nature of this binary. The term ‘western’ is also employed to broadly characterise knowledge production rooted in Euro-American intellectual traditions, while recognising that these traditions were also informed by thought and events emerging from elsewhere in the world.

2 In Kinyarwanda: ‘Ntawubara ijoro kurusha uwariraye’.

3 Rwandan proverb which translates literally as the sadness of the chicken is known by its place of hunting, which means metaphorically that every person knows their difficulties and the way of coping with them.

4 Rwandan proverb which means a child is a leader.

5 Both projects are part of the Changing the Story network, which has the overarching aim of evaluating present and informing future civil society organisations work with, and for, young people in ‘post-conflict’ countries. See declarations section for further information.

6 Rwandan proverb which means ‘the proverb reflects reality’.

7 An earlier version of the reflections on proverbs appeared as a blog (Uwihoreye and Pells 2020).

8 Huye is a district in the south of Rwanda.

9 Kabera is the producer, screenwriter, and director of numerous documentaries and films related to the 1994 Genocide Against the Tutsi and its legacies including *100 Days* (2001), *Keepers of Memory* (2004), *Africa United* (2010), and *Intore* (2014).

10 Rwandan proverb translated literally means what destroys the navel does not escape the intestine and metaphorically means the consequences of problems experienced by parents are also shared to their descendants.
